# Virtual reality immersion compared to monitored anesthesia care for hand surgery: A randomized controlled trial

**DOI:** 10.1371/journal.pone.0272030

**Published:** 2022-09-21

**Authors:** Adeel A. Faruki, Thy B. Nguyen, Doris-Vanessa Gasangwa, Nadav Levy, Sam Proeschel, Jessica Yu, Victoria Ip, Marie McGourty, Galina Korsunsky, Victor Novack, Ariel L. Mueller, Valerie Banner-Goodspeed, Tamara D. Rozental, Brian P. O’Gara

**Affiliations:** 1 Department of Anesthesiology, University of Colorado Hospital, Aurora, CO, United States of America; 2 University of Colorado Medical School, Aurora, CO, United States of America; 3 St. George’s University School of Medicine Grenada, St. George’s, West Indies, Grenada; 4 Beth Israel Deaconess Medical Center Department of Anesthesia, Critical Care, and Pain Medicine, Harvard Medical School, Boston, MA, United States of America; 5 Case Western Reserve University School of Medicine, Cleveland, Ohio, United States of America; 6 Nova Southeastern School of Osteopathic Medicine, Fort Lauderdale, FL, United States of America; 7 University of Massachusetts, Boston, MA, United States of America; 8 Department of Anesthesiology, Spectrum Healthcare Partners, Portland, ME, United States of America; 9 Research Authority and Clinical Research, Soroka University Medical Center, Beer-Sheva, Israel; 10 Anesthesia Research Center, Massachusetts General Hospital, Boston, MA, United States of America; 11 Division of Hand and Upper Extremity Surgery, Department of Orthopaedic Surgery, Beth Israel Deaconess Medical Center, Boston, MA, United States of America; Taipei Medical University, TAIWAN

## Abstract

**Introduction:**

Common anesthesia practice for hand surgery combines a preoperative regional anesthetic and intraoperative monitored anesthesia care (MAC). Despite adequate regional anesthesia, patients may receive doses of intraoperative sedatives which can result in oversedation and potentially avoidable complications. VR could prove to be a valuable tool for patients and providers by distracting the mind from processing noxious stimuli resulting in minimized sedative use and reduced risk of oversedation without negatively impacting patient satisfaction. Our hypothesis was that intraoperative VR use reduces sedative dosing during elective hand surgery without detracting from patient satisfaction as compared to a usual care control.

**Methods:**

Forty adults undergoing hand surgery were randomized to receive either intraoperative VR in addition to MAC, or usual MAC. Patients in both groups received preoperative regional anesthesia at provider discretion. Intraoperatively, the VR group viewed programming of their choice via a head-mounted display. The primary outcome was intraoperative propofol dose per hour (mg · hr^-1^). Secondary outcomes included patient reported pain and anxiety, overall satisfaction, functional outcome, and post anesthesia care unit (PACU) length of stay (LOS).

**Results:**

Of the 40 enrolled patients, 34 completed the perioperative portion of the trial. VR group patients received significantly less propofol per hour than the control group (Mean (±SD): 125.3 (±296.0) vs 750.6 (±334.6) mg · hr^-1^, p<0.001). There were no significant differences between groups in patient reported overall satisfaction, (0–100 scale, Median (IQR) 92 (77–100) vs 100 (100–100), VR vs control, p = 0.087). There were no significant differences between groups in PACU pain scores, perioperative opioid analgesic dose, or in postoperative functional outcome. PACU LOS was significantly decreased in the VR group (53.0 (43.0–72.0) vs 75.0 (57.5–89.0) min, p = 0.018).

**Conclusion:**

VR immersion during hand surgery led to significant reductions in intraoperative propofol dose and PACU LOS without negatively impacting key patient reported outcomes.

## Introduction

Over 600,000 wrist and hand surgeries were performed in the ambulatory setting in the United States in 2006 and trends indicate that this figure will grow for years to come [1, 2]. In England, there is a projected increase of as much as 75% for common elective hand operations such as trigger finger release in the coming decades [3]. With this increased volume, a focus on outpatient surgery, and an aging surgical population, a focus for optimizing care for these patients will undoubtedly involve modification to anesthetic practices. One of the most common approaches of anesthesia for hand surgery is the combination of regional anesthesia and monitored anesthesia care (MAC) [4, 5]. By inhibiting both motor and sensory function, regional anesthesia provides optimal surgical conditions and effective analgesia throughout the perioperative period. With an adequate regional anesthetic, patients undergoing hand surgery would ideally only require intraoperative sedation for anxiolysis. Commonly, however, patients undergoing MAC receive doses of intravenous anesthetics such as propofol which may be out of proportion to their requirements for anxiolysis. This can increase the risk of oversedation leading to hypotension, upper airway obstruction, apnea, and postoperative neurocognitive disorders [6–8]. Such excessive sedation can result in serious morbidity. In fact, twenty-one percent of MAC claims in the Closed Claims Database were related to respiratory depression due to an overdose of sedative medications [9]. Therefore, an intervention that could reduce intraoperative sedative dosing could be valuable in preventing adverse events. However, simply providing less sedatives is not a management strategy likely to be equally valued by patients and physicians, especially if it comes at the expense of comfort and satisfaction.

Virtual reality (VR) offers a potential means for anxiolysis without the use of sedatives. VR use has expanded from the entertainment sector to the fields of medical education, rehabilitation, and the management of mental health and chronic pain [10]. VR’s purported benefit in the management of patients with pain or anxiety is through providing an immersive experience capable of distracting the mind from processing noxious stimuli. Although VR has been shown to provide effective anxiolysis for minor procedures such as endoscopy and dressing changes, currently there is only limited evidence to support VR’s effectiveness during surgery [11–14]. Given this, we conducted a randomized, controlled clinical trial with the objective of investigating whether VR immersion could reduce the amount of sedatives administered during hand surgery with regional anesthesia and MAC as compared to MAC alone. Our hypothesis was that intraoperative VR use reduces sedative dosing during elective hand surgery without detracting from patient satisfaction as compared to a usual care control.

## Methods

### Design

**Fig 1:**
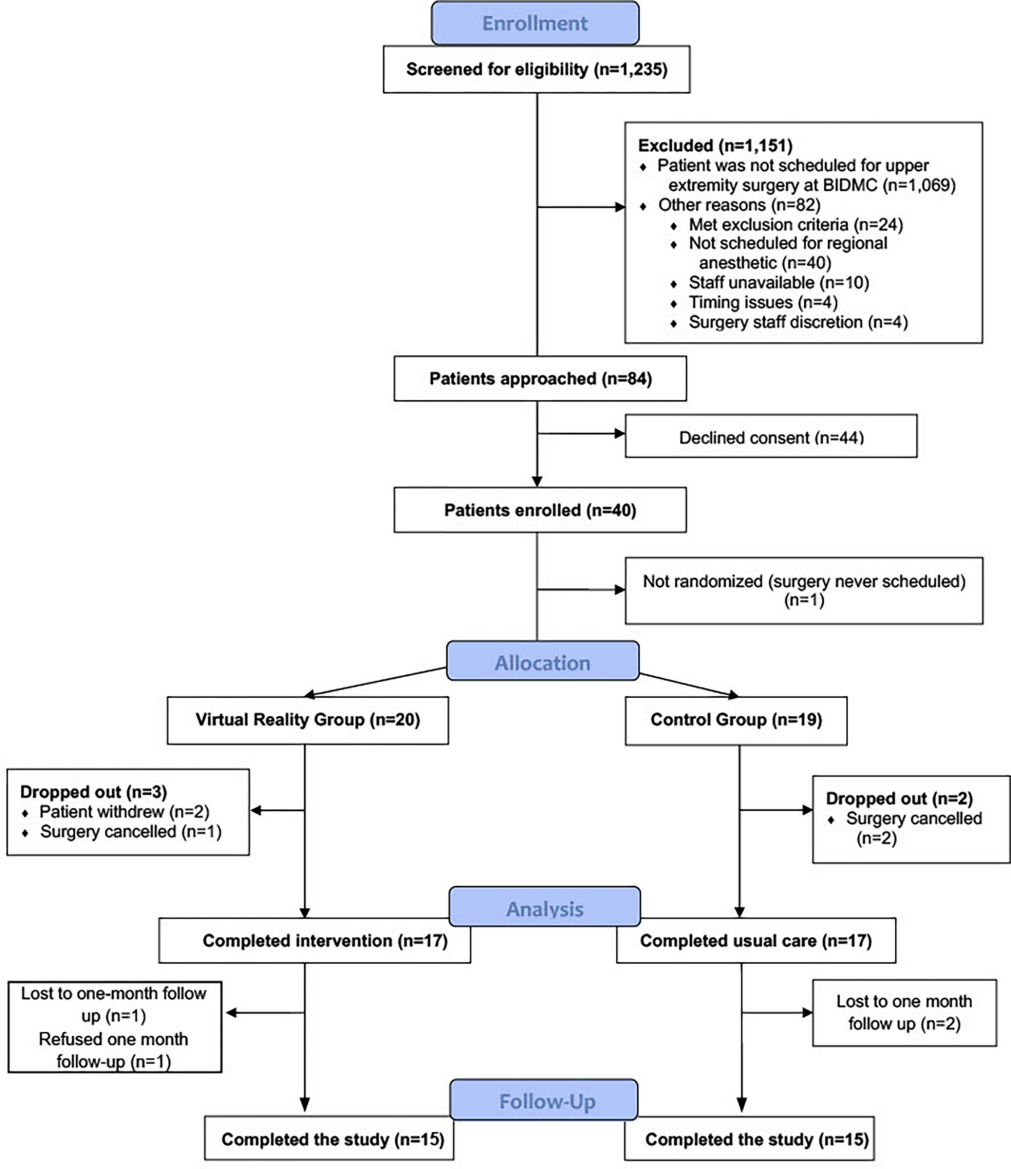
Consolidated Standards of Reporting Trials (CONSORT) flow diagram.

**Fig 2:**
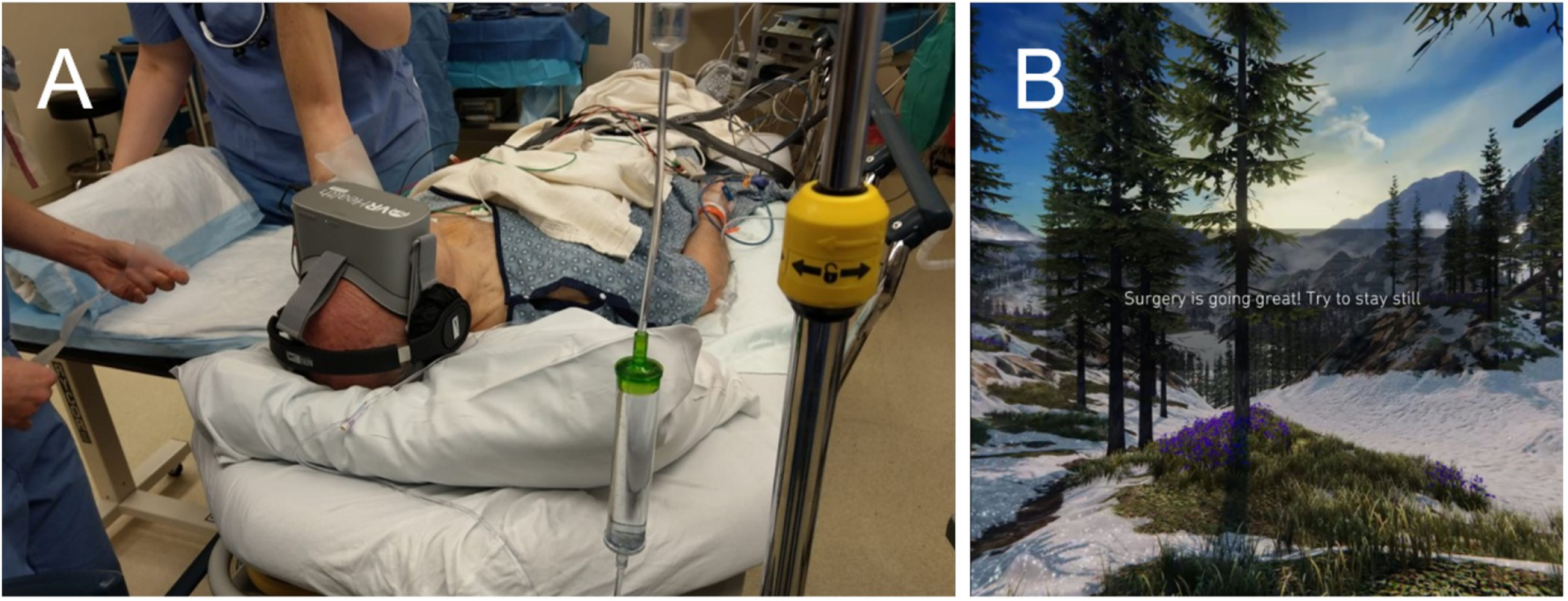
A) Image of a study patient using the VR equipment. B) Screenshot of a typical immersive environment with an example of text communication from study personnel. The individual in this manuscript has given written informed consent (as outlined in PLOS consent form) to publish this image.

This prospective, open-label, randomized single center study with a two-arm parallel group design was conducted at Beth Israel Deaconess Medical Center (BIDMC). This study was approved by the University’s Institutional Review Board (IRB #2018-P000398) and written informed consent was obtained from all subjects participating in the trial. The individual whose image in seen in Fig 2 has given written informed consent (as outlined in PLOS consent form) to publish these case details. The trial was registered prior to patient enrollment at clinicaltrials.gov (NCT03614325. Principal investigator: Brian O’Gara, Date of registration: August 3, 2018). The clinical trial was designed and executed following the Consolidated Standards of Reporting Trials (CONSORT) guidelines and the Standard Protocols Items: Recommendations for Interventional Trials (SPIRIT) checklist (Fig 1). The study had no interim analysis, trial audit or adaptation performed.

### Study population

Screening for eligible patients was performed using hand surgery clinic visit referral lists at BIDMC. Patients were eligible for inclusion if they were an adult (age 18+) scheduled for hand surgery with regional anesthesia. We excluded patients who were less than 18 years of age, were undergoing general anesthesia for the procedure, had an active infection or open wound of the face or eye area, had a history of seizures or epilepsy, planned to wear hearing aids intraoperatively, had a pacemaker or other implanted medical device, were on droplet or airborne precautions, did not speak English, or were likely to require heavy sedation to facilitate a more invasive surgery according to the surgeon. Eligible patients who provided written, informed consent were prospectively randomized in a 1:1 allocation with block size of 2 using the Research Electronic Data Capture (REDCap) randomization module. Randomization was not stratified, and the study statistician carried out the randomization. Randomization sequence and block size was concealed from investigators during the study. REDCap is a HIPAA compliant, web-based application designed to securely and anonymously store data capture [15]. Due to the nature of the intervention, investigators, participants, and care providers were not blinded to treatment allocation.

### Intervention: Virtual reality immersive relaxation

Patients randomized to the VR immersion group viewed the programming of their choice via an Oculus Go Standalone MH-A64 VR head-mounted display (Irvine, CA, USA) with Altec Lansing MZX667-BLK Evolution2 noise cancelling headphones (New York, NY USA) for the length of the procedure. The software used was VRReliever Version 0.3.4 from XRHealth (Tel Aviv, Israel). The program has been updated by XRHealth and the comparable application is now named “PD-810 Mindset.” Patients could select from several immersive 360-degree VR environments in the “Explore Experience” designed to promote relaxation and calmness, such as a peaceful meadow, forest or mountaintop (Fig 2). Patients could also listen to guided meditation in the immersive environments or select from a library of videos on a web-based user interface which was displayed as a theatre screen surrounded by a “starry sky” background. Patients were allowed to switch between programs during surgery, with assistance from study personnel monitoring the experience in parallel with a tablet (Samsung Galaxy Tab A SM-T590, Seoul, South Korea). Supplemental anesthetics and/or analgesics could be administered either upon patient request or at the discretion of the anesthesia provider according to their clinical judgement. The VR immersion program ran for the duration of the procedure. At the end of the procedure, the headset and headphones were removed, and standard postoperative care commenced.

### Control group

Control patients underwent MAC as determined by their anesthesia provider without the addition of the VR headset or headphones. No investigators were ever assigned to be the anesthesia provider of study patients in either group. Study team members were not present in the operating room to conduct any research activities for control group patients.

### Clinical management in both groups

Preoperatively, patients received a brachial plexus nerve block at the discretion of the preoperative regional anesthesia team. All intraoperative anesthetic management was at the discretion of the treating anesthesia provider. The intraoperative anesthetic for patients in either group was not formally protocolized, but it should be noted that at our institution MAC typically and overwhelmingly consists of a titrated propofol infusion. Patients were monitored (i.e. vital signs, capnography) according to American Society of Anesthesiologists’ standards. Postoperative care including PACU management was conducted according to current BIDMC standards.

### Variables and outcome measurements

Data on preoperative anesthetic management, intraoperative anesthetic and analgesic dosing, surgical characteristics, intraoperative vital signs and level of airway assistance, other intraoperative medications administered, length of PACU stay, postoperative analgesia administration, and postoperative pain scores were abstracted from electronic medical records. Demographic data (including, but not limited to age, race, sex, BMI, and comorbidities) was also collected. The primary outcome of this study was intraoperative propofol dose per hour (expressed as mg hr^-1^). Analysis of propofol dose accounting for the length of the procedure was necessary as the duration of the procedures were not consistent between patients. Secondary outcomes included patient reported control of pain and anxiety, and overall satisfaction. For these outcomes, participants were interviewed in the PACU by study personnel and were asked to report their level of agreement with statements such as “My pain was well controlled” and “I felt anxious” via quantifiable slide bars with a scale from 0–100. The full survey is available in the S2 File. Additional secondary outcomes included intraoperative airway characteristics (obstructed breathing, requirement for an airway adjunct, etc), PACU length of stay (LOS), postoperative analgesics administered and postoperative pain scores. Lastly, the Disabilities of the Arm, Shoulder and Hand (DASH) questionnaire, an instrument designed to measure hand disability, was used to assess functional outcome. The DASH score is a validated patient reported outcome measure which determines a person’s level of hand disability on a 0–100 scale, with 100 indicating the most severe disability [16]. Pre-operative *Quick*DASH scores were retrospectively collected from the surgical preoperative assessment to a compare between pre- and post-operative disability scores. Postoperative DASH scores were obtained by study personnel via telephone at the time of the surgical follow up visit. To better compare the pre and post scores, the DASH scores were converted *post hoc* to *Quick*DASH scores using only the common items assessed in both instruments. No binary outcomes were assessed in this study.

### Statistical analysis

A modified intention to treat approach was used. We defined the modified intention to treat population to include all patients who underwent the surgical procedure and did not withdraw consent prior to the surgery. Therefore, we have excluded from the analysis 4 subjects in whom the surgery was never performed (3 subjects from Control group and 1 from VR group) and two subjects who withdrew consent (both from VR group). Normality assessment was performed by evaluating the distribution of the variables on histogram and Q-Q plots. We analyzed the differences between the groups in the medication dosages with a t-test. Additionally, a sensitivity analysis with a bootstrapped t-test was performed to assess differences in propofol doses accounting for both the duration of the procedure and the patient’s weight (expressed as mg kg^-1^ min^-1^). The bootstrapped methodology with 5000 simulations was used to obtain a more robust inference after analyzing the distribution of the outcome, as many participants in the intervention group received zero propofol doses, in attempt of obtaining a more robust inference. Likewise, the proportion of patients administered any propofol was compared using Fisher’s exact test. Postoperative outcomes and the results of the PACU survey were analyzed by Mann-Whitney tests and the frequency at which the medications were administered were analyzed by Chi-square or Fisher’s exact tests.

We have presented binary data as n (%) and continuous data with medians and interquartile ranges (IQR). We used IBM SPSS Statistics Version 25 for the analysis and considered a two-sided p-value <0.05 to be statistically significant. The statistical analysis plan was approved by the authors prior to the collection of results and was previously published in a methods manuscript [17]. No interim analysis was performed.

### Sample size calculation

A previous randomized pilot trial studying VR use in the operating room for orthopedic surgery reported a >50% reduction in propofol dose as compared to control, although these findings were not statistically significant [13]. Based on this data, we conservatively anticipated that we may be able to observe a 30% reduction in intraoperative propofol dose between groups. Using a t-test with a two sided α of 0.05, 80% power, an anticipated propofol dose reduction of 30% for the intervention group, with mean propofol doses of 155 milligrams hr^-1^ (standard deviation ±45 mg · hr^-1^) and 108.5 mg hr^-1^ (SD ±45 mg hr^-1^) we anticipated needing a sample size of 32 patients. In anticipation of possible drop out, we added 8 patients to the calculated sample size with a total planned enrollment of 40 patients, with 20 patients in each group.

## Results

A total of 1235 patients were screened, of which 84 met eligibility criteria. 44 patients declined consent for the trial and 40 patients were enrolled between December 2018 and August 2019 (Fig 1). A total of 34 patients (17 in each group) completed the study protocol. Four of the enrolled patients did not have their surgery performed and 2 patients withdrew from the study. Of the 34 patients who completed the study protocol, two patients in the control group and one patient in the VR group were lost to follow-up and were not administered the one-month postoperative DASH questionnaire. One patient in the VR group refused to participate in the questionnaire. The patient who refused the follow-up questionnaire still agreed to have their data analyzed. The data from these patients were included in the analysis of perioperative outcomes. Follow-up was completed by December 2019 and the trial was then concluded. Baseline demographics, medical comorbidities, and surgical characteristics were similar between both groups with the exception of imbalances in the previous use of VR, the duration of surgery, dose of mepivacaine administered during the preoperative regional anesthetic, and the number of patients receiving midazolam during the perioperative period (Table 1). These imbalances did not appear to have a consistent directionality favoring one group or the other and were deemed unlikely to bias the overall results away from the null. The majority of patients in both groups underwent preoperative brachial plexus block using bupivacaine and/or mepivacaine. Two patients from the VR group and one patient from the control group did not receive a preoperative regional anesthetic due to booking error or regional anesthesia staff unavailability and thus were given a local block by the surgeon.

**Table 1:** Preoperative characteristics.

	Control (n = 17)		Virtual Reality (n = 17)	Standardized Difference*
Age, years	50.1±11.1		48.7±19.0	-0.09
Female sex	8 (47.1)		5 (29.4)	-0.37
BMI, kg/m^2^	28.7±5.2		28.1±4.8	-0.12
Diabetes	4 (23.5)		2 (11.8)	-0.31
Hypertension	6 (35.3)		9 (52.9)	0.36
Dyslipidemia	6 (35.3)		5 (29.4)	-0.13
Obesity	7 (41.2)		3 (17.6)	-0.53
COPD	4 (23.5)		3 (17.6)	-0.15
Depression	6 (35.3)		3 (17.6)	-0.41
Anxiety	3 (17.6)		2 (11.8)	-0.17
*Previous Use of VR*				
Never,	13 (76.5)		10 (58.8)	0.74
Once	2 (11.8)		3 (17.6)	
A few times	1 (5.9)		4 (23.5)	
Many times	0 (0)		1 (5.9)	
*Procedure Type* ^a^				
Carpal tunnel		4 (23.5)	4 (23.5)	0.00
Dupuytren’s contracture		1 (5.9)	2 (11.8)	0.21
Tendon/ligament repair		5 (29.4)	3 (17.6)	-0.28
Ganglion removal		3 (17.6)	3 (17.6)	0.00
Ulnar neurolysis/decompression		2 (11.8)	4 (23.5)	0.31
Mass/cyst excision		3 (17.6)	1 (5.9)	-0.37
Reduction and/or internal fixation		2 (11.8)	3 (17.6)	0.17
Other		4 (23.5)	2 (11.8)	-0.31
Surgery length, minutes		27.0 (16.5–45.5)	19.0 (12.5–51.0)	-1.17
*Regional Anesthetic*				
Supraclavicular	4 (23.5)		7 (41.2)	0.38
Infraclavicular	11 (64.7)		8 (47.1)	-0.36
None/data missing	2 (11.8)		2 (11.8)	-0.35
Bupivacaine, mg	100 (75–100)		100 (67.5–100)	-0.21
Mepivacaine, mg	240 (200–300)		200 (200–200)	-1.17
Midazolam (preoperative/ intraoperative)	17 (100)		13 (76.5)	-0.78

Values are presented as n (%), mean ±SD, or median (Q1-Q3) depending on variable type and distribution.

^a^ Multiple bookings per case are possible.

^b^ Calculated as the difference in means or proportions divided by the pooled standard deviation. * Significant imbalance is defined as an absolute standardized difference > 0.67.

### Primary outcome: Intraoperative propofol dose per hour

**Fig 3:**
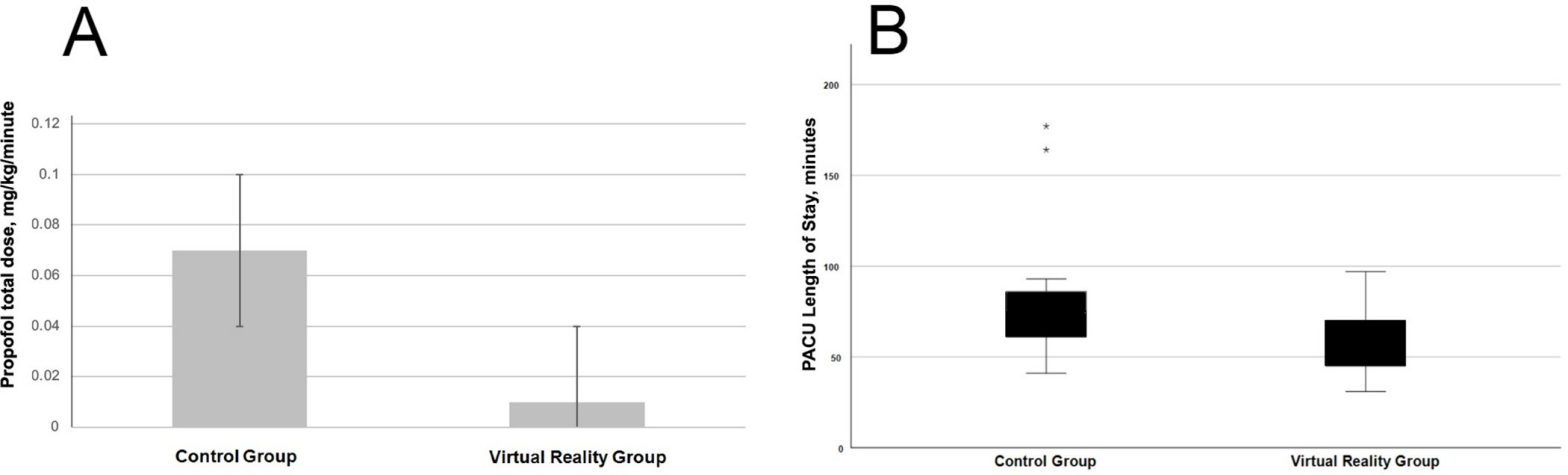
A) Propofol mean total dose by group B) PACU median length of stay by group.

Patients in the VR group received significantly less propofol per hour than those in the control group (Mean (±SD) VR 125.3 (±296.0) vs control 750.6 (±334.6) mg hr^-1^, p<0.001) (Fig 3). VR group patients also received significantly less propofol as defined in terms of total dose, bolus dose, and infusion dose (Table 2). A sensitivity analysis for mean total propofol dose relative to length of the procedure and patient’s weight (expressed as mg kg^-1^ min^-1^) also showed less propofol was used in the VR group than in the control group (Mean (±SD) 95%CI VR 0.01 (0.03).

**Table 2:** Intraoperative anesthetics administered by group.

	Control (n = 17)	Virtual Reality (n = 17)	*p Value*
Propofol	17/17 (100)	4/17 (23.5)	<0.001
Propofol bolus, mg	50 (52.5) 28.5–76.8	19.4 (45.9) 3.0–44.0	0.084
Propofol infusion, mg	306.7 (182.2) 229.6–397.3	74.7 (261.0) 0.0–213.6	0.006
Propofol total dose, mg	356.7 (216.7) 267.4–467.2	94.1 (304.1) 6.8–257.5	0.008
Propofol total dose, mg hr^-1^ ^a^	750.6 (334.6) 598.6–911.5	125.3 (296.0) 10.3–267.9	<0.001
Propofol total dose, mg kg^-1^ min^-1 b^	0.07 (0.03) 0.06–0.09	0.01 (0.03) 0.00–0.03	<0.001
Ketorolac	4/17 (23.5)	2/17 (11.7)	0.66

Values are presented as n (%) or mean (SD) 95%CI depending on variable type.

^a^ Primary outcome.

^b^ Sensitivity analysis.

0.00–0.03 vs control 0.07 (0.03) 0.06–0.09 mg kg^-1^ min^-1^, p-value based on a bootstrapped test <0.001). Of note, only 4/17 patients in the VR group received propofol intraoperatively, whereas every control patient received propofol (Fisher exact test p-value <0.001). Two of the VR patients who received propofol did not undergo a preoperative regional anesthetic. Of the remaining two patients in the VR group who received propofol, one was found to have an inadequate nerve block at the time of incision, and one patient experienced tourniquet pain during a prolonged operation. All patients in the control group received midazolam either with regional anesthesia or intraoperatively whereas 13/17 patients received midazolam (standardized difference = -0.78). Although there were no significant differences found in intraoperative intravenous ketamine, fentanyl, or ketorolac use between groups, significantly more patients in the VR group received supplemental local anesthesia by the surgeon (7/17 (41.2%) vs 1/17 (5.9%), p = 0.04).

### Patient reported outcomes

Results of the analysis of patient reported outcomes revealed no significant difference in overall satisfaction between groups (0–100 scale, Median (IQR) 92 (77–100) vs 100 (100–100), VR vs control, p = 0.087) (Table 3). Additionally, no significant differences were found in the level of agreement (0–100) with the prompted statements: “My pain was well-controlled during surgery” (100 (76–100) vs 100 (10–100), p = 0.12), “I felt relaxed during my procedure” (99 (79–100) vs 100 (100–100), p = 0.06), and “I felt anxious during my procedure” (0 (0–30) vs 0 (0–2), p = 0.68). Notably, the patients in the treatment group had a median 97% (IQR 79–100) agreement with the statement “I remember being aware of how I felt while I was in the operating room” in comparison to 0% (IQR 0–0) (p<0.001) in the control group. The one-month postoperative *Quick*DASH scores were not statistically significant between VR and control groups (20.5 (9.1–40.9) vs 40.9 (27.3–59.1), p = 0.056). A *post hoc* analysis did not reveal a significant difference between pre- and post-operative *Quick*DASH scores between groups (-7.9 (-21.1–1.2) vs 4.3 (-11.4–16.9), VR vs control, p = 0.22).

**Table 3:** Postoperative outcomes.

	Control (n = 17)	Virtual Reality (n = 17)	*p Value*
Overall satisfaction (scale 0–100)	100 (100–100)	92 (77–100)	0.09
*Agreement with the following statement (0–100)*:			
My pain was controlled	100 (100–100)	100 (76–100)	0.12
I felt relaxed	100 (100–100)	99 (79–100)	0.06
I felt anxious	0 (0–2)	0 (0–30)	0.36
I remember being aware of how I felt in the OR	0 (0–0)	97 (79–100)	<0.001
I would be interested in VR for future surgery	100 (68–100)	100 (69–100)	0.68
PACU pain score (0–10 scale) ^a^	0 (0–0)	0 (0–0)	0.33
Postoperative QuickDASH score ^b^	40.9 (27.3–59.1)	20.5 (9.1–40.9)	0.056
Change in QuickDASH score (post-pre)	4.3 (-11.4–16.9)	-7.9 (-21.1–1.2)	0.22
PACU Length of stay, min	75.0 (57.5–89.0)	53.0 (43.0–72.0)	0.018

Values are presented as n(%) or median (Q1-Q3) depending on variable type.

^a^ Multiple measurements per patient.

^b^ The QuickDASH is an 11-item questionnaire regarding hand disability. Higher scores indicate worse disability.

### Other secondary outcomes

There were no significant differences found between groups with regards to perioperative opioid analgesics administered or PACU pain scores. Intraoperative airway and respiration comments were documented in 14/17 and 15/17 patients in the VR and control groups, respectively. Respirations were characterized as normal with no need for assistance in 13/17 patients in both groups. One patient in the VR group required an oral airway, and one patient in the control group required a nasal trumpet for upper airway obstruction. PACU LOS was significantly decreased in the VR group compared to the control group (Median (IQR) VR 53.0 (43.0–72.0) vs control 75.0 (57.5–89.0) min, p = 0.018) (Table 3, Fig 3). No exploratory analyses were performed on this data. No unintended harm or effects occurred in either group during the study.

## Discussion

In this randomized controlled trial, patients randomized to VR immersion during hand surgery received significantly less intraoperative propofol compared to a usual care control without significant differences in patient-reported control of pain and anxiety, or overall satisfaction. Our trial is novel in that it is the first to report a significant reduction in sedative dosing with VR immersion during hand surgery in adults. To date, only two other studies have evaluated the effectiveness of VR immersion to reduce sedation dosing during surgery [13, 14]. Both studies were conducted in patients undergoing hip and knee arthroplasty, and neither reported significant reductions in propofol dose between VR patients and controls using either an anesthesiologist-provided or patient-controlled propofol infusion. Another strength of our study is the inclusion of both objective and patient-reported measures as well as functional outcomes to provide a comprehensive assessment of VR’s impact on the perioperative experience.

Our results revealed that patients in the VR group received an average of 125.3 mg hr^-1^ or median of 260 mg less propofol per case than patients in the usual care control group. While this reduction in dose is sizeable and has value as a surrogate marker for oversedation, we were unable to demonstrate that this reduction reduced the need for airway assistance per group. In this population, differences in serious but relatively rare adverse outcomes from over-sedation such as unplanned intubation or aspiration would need to be defined using a much larger sample. Given such feasibility issues, we opted to focus on the surrogative marker of propofol dose as the first step in investigating VR’s potential to reduce complications from oversedation. To more accurately analyze our primary outcome, we performed a sensitivity analysis to assess mean total propofol dose relative to the duration of the procedure and patient weight with a bootstrapped t-test which also indicated that the VR group received significantly reduced doses of intraoperative propofol in comparison to usual care control group. The infrequent need for propofol and frequent need for supplemental local anesthetics in the VR group reflects the importance of an adequate regional or local anesthetic when using VR as an adjunct to anesthesia. Specifically, awake patients using VR may require careful assurance of an adequate regional anesthetic before surgical incision and supplementation with additional local blocks as needed. Conversely, the higher dose of propofol administered to the control group may have been advantageous in supplementing possible incomplete regional blockade.

Despite receiving significantly less intraoperative propofol sedation, VR group patients did not have any significant differences compared to usual care group patients in self-reported assessments of pain, relaxation, anxiety, or overall satisfaction. Although we acknowledge that lack of a significant difference does not infer equivalence, this combination of findings represents an outcome that is important to both patients and providers: using VR immersion, potentially harmful or unnecessary sedation can be avoided without compromising patient comfort during hand surgery. Furthermore, we observed that VR group patients were discharged from the PACU 22 minutes earlier than control patients. At our institution, there are typically 10–12 upper extremity surgeries booked per day. Therefore, a reduction in PACU length of stay of 22 minutes per case could help optimize perioperative efficiency if the technique were to be used more widely. Finally, in our assessment of postoperative functional recovery via the *Quick*DASH questionnaire, a 20-point difference was observed favoring the VR group but this finding was not statistically significant. As noted above, the minimum clinically important difference for the *Quick*DASH is 10 points, therefore this finding could have potential implications for long term postoperative recovery should it be found to be significant in future trials [18].

A recent meta-analysis of VR use for acute and chronic pain syndromes highlighted several methodological issues limiting the interpretation of VR clinical trials [19]. Our trial shares many of these limitations. Study patients were aware of the possibility of receiving reduced doses of sedatives by being enrolled in this trial. Such willingness for minimal sedation may not be generalizable to patients who prefer to be sedated and could have affected the results of our patient-reported outcomes through selection bias. The second major limitation of this study is that providers in the study were not blinded. We decided against blinding for a couple of reasons. First, our intention was to compare VR immersion to a usual care control so that our results would be generalizable to the typical anesthetic experience for hand surgery. We felt the results of this comparison would be more meaningful to practicing clinicians rather than by using a comparison group which is not representative of typical anesthetic management. Second, we felt the necessary communication regarding program selection between patients and study members during the procedure would be a constant source of unblinding which would be difficult to fully eliminate. However, the lack of blinding of providers could have contributed to the dramatic differences between propofol dosing between groups. Because of this, we believe it is also important to take into consideration the results of the patient surveys indicating no significant differences between groups in control of pain or anxiety, or in overall satisfaction. However, due to the nature of the intervention, it is not possible to blind the participants to the group assignment. Therefore, the responses to the survey could have been influenced by the patient’s perception that VR would be effective. Because of the potential for bias to influence both of these outcomes, our results should be interpreted as preliminary and needing validation in future trials. Further, given these major limitations our results are therefore best suited to describe how the incorporation of VR immersion into current anesthesia practice for hand surgery can compare with the standard of care, not to serve as proof that VR is an effective pain control modality or is superior to other distraction techniques. Such determinations would require carefully blinded studies which also attempt to control for the placebo effect. Understanding these limitations, we encourage readers to examine these results with caution and in the context of knowing the unblinded design. Lastly, our intervention included multiple programs in the interest of offering variety and customizability, rather than focusing on a specific VR modality like environmental immersion. Therefore, our results do not demonstrate which particular VR experience is most effective for this indication.

In conclusion, our results suggest that VR immersion can reduce intraoperative propofol dosing and PACU LOS without negatively impacting patient satisfaction during hand surgery with regional anesthesia. VR use in similar clinical contexts could potentially minimize sedative-related perioperative complications in vulnerable patients and is worthy of further investigation.

## Supporting information

S1 File CONSORT checklist. (DOC)

S2 File Patient questionnaires. (PDF)

S3 File Data export. (CSV)

S4 File Protocol explanation letter. (PDF)

S5 File Original master protocol. (PDF)

## References

[1] Jain NB, Higgins LD, Losina E, Collins J, Blazar PE, Katz JN. Epidemiology of musculoskeletal upper extremity ambulatory surgery in the United States. BMC Musculoskelet Disord. 2014;15(1). doi: 10.1186/1471-2474-15-4 24397703 PMC3893587

[2] Hollenbeck BK, Dunn RL, Suskind AM, Zhang Y, Hollingsworth JM, Birkmeyer JD. Ambulatory surgery centers and outpatient procedure use among medicare beneficiaries. Med Care. 2014;52(10):926–31. doi: 10.1097/MLR.0000000000000213 25185636 PMC4163137

[3] Bebbington E, Furniss D. Linear regression analysis of Hospital Episode Statistics predicts a large increase in demand for elective hand surgery in England. J Plast Reconstr Aesthetic Surg [Internet]. 2015;68(2):243–51. Available from: doi: 10.1016/j.bjps.2014.10.011 25455287 PMC4315884

[4] Fingerman M. Regional anesthesia for outpatient hand surgery: Ultrasound-guided peripheral nerve block. J Hand Surg Am [Internet]. 2011;36(3):532–4. Available from: doi: 10.1016/j.jhsa.2010.11.037 21277701

[5] Vallera C, Laporte DM. Monitored anesthesia care for hand surgery in adults. J Hand Surg Am [Internet]. 2011;36(7):1235–6. Available from: doi: 10.1016/j.jhsa.2011.01.040 21497026

[6] De Wit F, Van Vliet AL, De Wilde RB, Jansen JR, Vuyk J, Aarts LP, et al. The effect of propofol on haemodynamics: cardiac output, venous return, mean systemic filling pressure, and vascular resistances. Br J Anaesth [Internet]. 2016;116(6):784–9. Available from: doi: 10.1093/bja/aew126 27199311

[7] Lodenius Å, Ebberyd A, Hårdemark Cedborg A, Hagel E, Mkrtchian S, Christensson E, et al. Sedation with Dexmedetomidine or Propofol Impairs Hypoxic Control of Breathing in Healthy Male Volunteers. Anesthesiology. 2016;125(4):700–15. 27483127 10.1097/ALN.0000000000001236

[8] Sieber FE, Zakriya KJ, Gottschalk A, Blute MR, Lee HB, Rosenberg PB, et al. Sedation depth during spinal anesthesia and the development of postoperative delirium in elderly patients undergoing hip fracture repair. Mayo Clin Proc [Internet]. 2010;85(1):18–26. Available from: doi: 10.4065/mcp.2009.0469 20042557 PMC2800291

[9] Bhananker SM, Posner KL, Cheney FW, Caplan RA, Lee LA, Domino KB. Injury and liability associated with monitored anesthesia care: A closed claims analysis. Anesthesiology. 2006;104(2):228–34. doi: 10.1097/00000542-200602000-00005 16436839

[10] Pourmand A, Davis S, Lee D, Barber S, Sikka N. Emerging Utility of Virtual Reality as a Multidisciplinary Tool in Clinical Medicine. Games Health J. 2017;6(5):263–70. doi: 10.1089/g4h.2017.0046 28759254

[11] Vázquez J, Wiederhold B, Miller I, Wiederhold M. Virtual Reality Assisted Anaesthesia During Upper Gastrointestinal Endoscopy: Report of 115 Cases. PdfsSemanticscholarOrg [Internet]. 2017;(January):75–82. Available from: https://pdfs.semanticscholar.org/f4d7/9089a6d2082061040311ce69d1d52934bdbb.pdf

[12] Morris LD, Louw QA, Grimmer-Somers K. The effectiveness of virtual reality on reducing pain and anxiety in burn injury patients: A systematic review. Clin J Pain [Internet]. 2009 Nov [cited 2018 Nov 28];25(9):815–26. Available from: http://www.ncbi.nlm.nih.gov/pubmed/19851164 doi: 10.1097/AJP.0b013e3181aaa909 19851164

[13] Chan PY, Scharf S. Virtual Reality as an Adjunctive Nonpharmacological Sedative during Orthopedic Surgery under Regional Anesthesia: A Pilot and Feasibility Study. Anesth Analg. 2017;125(4):1200–2. doi: 10.1213/ANE.0000000000002169 28598921

[14] Huang MY, Scharf S, Chan PY. Effects of immersive virtual reality therapy on intravenous patient-controlled sedation during orthopaedic surgery under regional anesthesia: A randomized controlled trial. PLoS One. 2020;15(2):1–12. doi: 10.1371/journal.pone.0229320 32092098 PMC7039521

[15] Harris PA, Taylor R, Thielke R, Payne J, Gonzalez N, Conde JG. Research Electronic Data Capture (REDCap)—A metadata-driven methodology and workflow process for providing translational research informatics support. J Biomed Inf. 2009;11(2):687–701. doi: 10.1016/j.jbi.2008.08.010 18929686 PMC2700030

[16] Hudak PL, Amadio PC, Bombardier C. Development of an upper extremity outcome measure: The DASH (disabilities of the arm, shoulder, and head). Am J Ind Med. 1996;29(6):602–8. 8773720 10.1002/(SICI)1097-0274(199606)29:6<602::AID-AJIM4>3.0.CO;2-L

[17] Faruki A, Nguyen T, Proeschel S, Levy N, Yu J, Ip V, et al. Virtual reality as an adjunct to anesthesia in the operating room. Trials. 2019;20(1):1–7. 31882015 10.1186/s13063-019-3922-2 PMC6935058

[18] Polson K, Reid Duncan D, McNair PJ, Larmer P. Responsiveness, minimal importance difference and minimal detectable change scores of the shortened disability arm shoulder hand (QuickDASH) questionnaire. Man Ther [Internet]. 2010 Aug [cited 2021 May 2];15(4):404–7. Available from: https://pubmed.ncbi.nlm.nih.gov/20434942/ doi: 10.1016/j.math.2010.03.008 20434942

[19] Chuan A, Zhou JJ, Hou RM, Stevens CJ, Bogdanovych A. Virtual reality for acute and chronic pain management in adult patients: a narrative review [Internet]. Vol. 76, Anaesthesia. Blackwell Publishing Ltd; 2021 [cited 2021 May 2]. p. 695–704. Available from: https://pubmed.ncbi.nlm.nih.gov/32720308/32720308 10.1111/anae.15202

