# Supplementary material for: Virtual reality immersion compared to monitored anesthesia care for hand surgery: A randomized controlled trial
Source: PLoS One. 2022 Sep 21;17(9):e0272030. doi: 10.1371/journal.pone.0272030 (PMC9491608; doi:10.1371/journal.pone.0272030)
Supplement: S2 File — (PDF) [file pone.0272030.s002.pdf]

Virtual Reality in the Operating Room: Using Immersive Relaxation as an Adjunct to Anesthesia

Baseline Survey

Thank you for your participation in our study. We would like to ask you a few questions.

Have you ever used a VR headset before?	◯ Never<br>◯ Once<br>◯ A few times<br>◯ Many times
Have you ever been interested in using a VR headset?	◯ Yes<br>◯ No<br>◯ I am unsure.
I think that VR should be used for patients undergoing hand or wrist surgery.	◯ True<br>◯ False<br>◯ Unsure

**Please respond to each item about your surgery by marking one box per row. In the past 7 days....**					
	Never	Rarely	Sometimes	Often	Always
I felt fearful.	◯	◯	◯	◯	◯
I found it hard to focus on anything other than my anxiety.	◯	◯	◯	◯	◯
My worries overwhelmed me.	◯	◯	◯	◯	◯
I felt uneasy.	◯	◯	◯	◯	◯
I felt nervous.	◯	◯	◯	◯	◯
I felt like I needed help for my anxiety.	◯	◯	◯	◯	◯
I felt anxious.	◯	◯	◯	◯	◯
I felt tense.	◯	◯	◯	◯	◯

Baseline Survey - Version Date: 03/20/2019

For Study Staff:

VRHealth Study ID: __________

Date and Time of Survey Administration: _________ (MM/DD/YYYY) at _____:_____

Person providing responses (circle one):
Patient
Surrogate: _______________ (relation to patient)

Person recording responses: ________________________

Additional notes (if applicable):

Virtual Reality in the Operating Room: Using Immersive Relaxation as an Adjunct to Anesthesia

Thank you for your participation in our study. We would like to ask you a few questions about your experience during the study.

Please indicate which group you were assigned to

◯ Virtual Reality
◯ Control Group

Please rate your agreement with the following:

I felt that the VR headset was comfortable.	Strongly Disagree	Neutral	Strongly Agree
	(Place a mark on the scale above)		
I enjoyed the selection of VR programs/environments.	Strongly Disagree	Neutral	Strongly Agree
	(Place a mark on the scale above)		
The VR program was easy to use.	Strongly Disagree	Neutral	Strongly Agree
	(Place a mark on the scale above)		
My pain level was controlled during surgery.	Strongly Disagree	Neutral	Strongly Agree
	(Place a mark on the scale above)		
I felt relaxed during my procedure.	Strongly Disagree	Neutral	Strongly Agree
	(Place a mark on the scale above)		
I felt anxious during my procedure.	Strongly Disagree	Neutral	Strongly Agree
	(Place a mark on the scale above)		
I felt nauseated during my procedure.	Strongly Disagree	Neutral	Strongly Agree
	(Place a mark on the scale above)		
I remember being aware of how I felt while I was in the operating room for my procedure.	Strongly Disagree	Neutral	Strongly Agree
	(Place a mark on the scale above)		
I would be interested in a VR program if I was going to have another surgery.	Strongly Disagree	Neutral	Strongly Agree
	(Place a mark on the scale above)		

I think that VR should be used for patients undergoing hand or wrist surgery.	◯ True ◯ False ◯ Unsure
Do you have any suggestions for environments or programs you might be interested in?	________________________________
Are there any aspects of this study that you would change for future participants?	☐ Increase the number of relaxation modules<br>☐ Change the type of VR headset used<br>☐ Other
If other, please describe what you might change	________________________________
Please rate your overall satisfaction with this study.	Strongly Disagree Neutral Strongly Agree<br>*(Place a mark on the scale above)*
Please indicate any other feedback you have for the research team	____

Patient Satisfaction Survey - Version Date: 03/20/2019

For Study Staff:

VRHealth Study ID: __________

Date and Time of Survey Administration: _________ (MM/DD/YYYY) at _____:_____

Person providing responses (circle one):
Patient
Surrogate: _______________ (relation to patient)

Person recording responses: ________________________

Additional notes (if applicable):
