# Supplementary material for: Virtual reality immersion compared to monitored anesthesia care for hand surgery: A randomized controlled trial
Source: PLoS One. 2022 Sep 21;17(9):e0272030. doi: 10.1371/journal.pone.0272030 (PMC9491608; doi:10.1371/journal.pone.0272030)
Supplement: S4 File — (PDF) [file pone.0272030.s004.pdf]

SUMMARY OF PROTOCOL AMENDMENTS

Date: **December 9, 2020**

Protocol Title: **Virtual Reality in the Operating Room: Using Immersive Relaxation as an Adjunct to Anesthesia**

Principal Investigator: **Brian O'Gara, M.D.**

Re: **Summary of Protocol Amendments**

This communication is to detail the changes that were been made to the Dr. O'Gara's "Virtual Reality in the Operating Room: Using Immersive Relaxation as an Adjunct to Anesthesia" protocol in the form of amendments while the study was active. The original IRB approved Master Protocol ("Part B") was dated 08/28/2018. Since then the following changes were made;

- **<u>Amendment approved 01/09/2019</u>** – Made the following changes to the protocol:
  - Expanded exclusion criteria to include patients who require deep sedation, and those deemed ineligible to approach by the surgeon
  - Added DASH outcome assessment to be completed 1 month (+/- 7 days) after surgery
  - Secondary outcomes updated to include the functional score from DASH assessment
- **<u>Amendment approved 02/20/2019</u>** – Made the following changes to the protocol:
  - Expanded exclusion criteria to include the history of vertigo
- **<u>Amendment approved 03/11/2019</u>** – Made the following changes to the protocol:
  - Removed history of motion sickness, history of claustrophobia, and history of vertigo from exclusion criteria
    - Moved to the risk section of the consent form
- **<u>Amendment approved 05/13/2019</u>** – Made the following changes to the protocol:
  - Updated Recruitment and Consent Procedures to state;
    - If research staff members are not able to approach a potentially eligible patient during their scheduled pre-operative appointment, the research team will call the patient to inform them about this research study. If subject express interest in participating, a research team member will approach the patient for written informed consent on the day of their surgery before medications are administered. Pre-screening may be

conducted over the phone. The process of obtaining informed consent will only conclude in-person. A script will be provided to outline how pre-screening would be conducted.

- ✧ **<u>Amendment approved 07/11/2019</u>** – Made the following changes to the protocol:
    - o Expanded inclusion criteria to include arm and elbow surgery in order to reflect the composition of upper extremity surgeries that will occur in the Division of Hand and Extremity Surgery
        - ▪ Reasoning being it would allow us to meet Specific Aim 1B to "determine whether the use of VR during upper extremity surgery can lead to improvements" in outcomes.
- ✧ **<u>Amendment approved 08/28/2019</u>** – Made the following changes to the protocol:
    - o Sample size justification clarified the propofol doses of 0.069 as "155mg/hr" (standard deviation 0.03 as "45 mg/hr") and 0.042 as "108.5 mg/hr" (SD 0.021 as "145 mg/hr"). The sample size total enrollment remains unchanged.
- ✧ **<u>Amendment approval 10/18/2019</u>** – Made the following changes to the protocol:
    - o "Dissemination of Research Results" updated to reflect that "if should a participant explicitly ask for study updates; subjects will be able to access research results after the study is completed and the findings are in print".

If you have any questions or would like any additional information, please do not hesitate to reach out.

Sincerely,

Brian O'Gara, MD
Beth Israel Deaconess Medical Center
Department of Anesthesia, Critical Care and Pain Medicine
Harvard Medical School
(p) 617-754-2129 • (e) bpogara@bidmc.harvard.edu
