# Supplementary material for: Virtual reality immersion compared to monitored anesthesia care for hand surgery: A randomized controlled trial
Source: PLoS One. 2022 Sep 21;17(9):e0272030. doi: 10.1371/journal.pone.0272030 (PMC9491608; doi:10.1371/journal.pone.0272030)
Supplement: S5 File — (PDF) [file pone.0272030.s005.pdf]

PART B
STUDY DESCRIPTION

**TITLE OF PROTOCOL**	**Virtual Reality in the Operating Room: Using Immersive Relaxation as an Adjunct to Anesthesia**
Principal Investigator	Brian O'Gara MD

B1. PURPOSE OF PROTOCOL

The purpose of this study is to investigate whether the use of virtual reality (VR) during wrist and hand surgery can reduce intraoperative pain and anxiety here at BIDMC through use of a randomized controlled trial. Specifically we aim to:

Specific Aim 1A: Evaluate whether the use of VR during wrist and hand surgery can lead to a reduction in intraoperative propofol dose, as compared to a usual care control.

Specific Aim 1B: Determine whether the use of VR during upper extremity surgery can lead to improvements in additional secondary outcomes, including length of postoperative anesthesia care unit (PACU) stay, postoperative pain scores and opioid doses.

Specific Aim 1C: Assess whether differences in patient satisfaction occur between individuals who do and do not receive VR during their hand/wrist procedure.

We hypothesize that the use of a virtual reality program during surgery can lead to a reduction in the dose of anesthesia needed for patient relaxation and comfort in the setting of hand and wrist operations with regional anesthesia.

B2. SIGNIFICANCE AND BACKGROUND FOR THE STUDY

There are nearly 600,000 ambulatory operations on the hand and wrist each year in the United States (Jain 2013). Currently, the standard anesthesia practice for these types of cases involves the combination of regional anesthesia (nerve block) and monitored anesthesia care (MAC). The goal of the regional anesthetic is to block both sensation and motor function to the operative area, which minimizes pain and discomfort for the patient and allows for good operating conditions for the surgeon. With an insensate and immobile extremity, the patient would ideally only require additional anesthetic agents to reduce intraoperative anxiety. In daily practice, however, such patients will commonly receive doses of intravenous sedatives, such as propofol, that most anesthesiologists would consider excessive. With the important role played by oversedation in the generation of postoperative adverse events, an intervention that could be shown to reduce intraoperative sedative requirements would prove to be very valuable (Bhananker 2006).

Virtual reality (VR) has expanded in recent years from the personal entertainment industry to the medical field. It has been found in preliminary study to be safe and effective as an adjunct to standard sedative and analgesic protocols in reducing pain and anxiety for patients undergoing upper gastrointestinal endoscopy, dental procedures, dressing changes for burns, and major orthopedic surgical cases (Vazquez 2017, Furman 2009, Morris 2009, Chan 2017). To date, the use of VR during hand and wrist surgery has not been studied to reduce intraoperative anesthetic requirements and improve the postoperative patient experience. A positive result from the proposed study could lead to a change in practice, with the introduction of a non-pharmacologic, patient-led intervention, potentially reducing the burden of unnecessary oversedation while providing a satisfactory perioperative experience.

B3. DESCRIPTION OF RESEARCH PROTOCOL

A. Study Design – Overview, Methods, Procedures

Study Overview
The proposed study is a randomized, controlled trial to evaluate the effectiveness of VR as an adjunct to standard anesthetic practice for hand and wrist surgery. Patients will be randomized to undergo immersion relaxation via the use of VR during their procedure or control. In both groups patients will undergo regional anesthesia preoperatively according to standard practice. Patients will then be assessed postoperatively to assess the intraoperative propofol dose between groups, as well as secondary outcomes including patient satisfaction.

Study Procedures

Patients who meet inclusion criteria with no exclusions will be approached to provide written informed consent in the perioperative period, as detailed below (Section B6). At this time we will provide patients with a study handout to ensure complete understanding of the study (see attached). We will also ask participants to complete a brief survey about how they are feeling at baseline (see attached). This should take about five minutes to complete.

After consent and prior to surgery, patients will be randomized in a 1:1 allocation to either intraoperative VR or usual care control. Given the nature of the intervention, we are unable to blind treatment allocation.

In both groups patients will undergo regional anesthesia preoperatively according to standard practice. Patients in the VR group will undergo immersion relaxation via the use of a VR headset and software packages for their intraoperative course as an adjunct to standard anesthesia practice. Control patients will undergo usual anesthesia care as determined by their anesthesia provider, without the addition of the VR device. All intraoperative anesthetic management and medication administration will be at the discretion of the treating provider. Patients will be monitored (vital signs, capnography) according to ASA standards for a MAC anesthetic. This data will be passively abstracted from the medical record in order to assess study outcomes.

It is possible that by virtue of being enrolled in this study participants may receive fewer medications than they would otherwise receive if they did not participate. For example, an anesthesia provider may not administer the initial bolus of pre-procedure propofol for a patient in the VR group, but instead continue to monitor them and only administer it if/when needed clinically. This potential for divergence from the standard process is well within clinically acceptable standards of care, but has been described as a potential risk associated with participation. In both groups patients will be monitored by their anesthesiologist, assessed and treated as necessary. The assessment is conducted by the clinical anesthesiologist independent of their participation in the study or study group assignment.

Intervention: Virtual Reality Immersive Relaxation

The intervention to be studied in the proposed trial is a relaxing, immersive virtual reality environment. Immersion takes place by wearing a VR headset connected to a software platform on an iPad. There are various environments that have been created with VR software, all designed to promote relaxation and calmness. Examples include sitting in a canoe on a river, a peaceful meadow or forest, or sitting on a mountaintop. To ensure comfort with the VR setting, patients in the intervention group will be allowed to select from one or several of the prespecified intraoperative relaxation packages, consistent of programs such as those mentioned above.

During their operation, after appropriate positioning has been taken place, the patient will don the VR headset and view the programming of their choice. Study team members will be available intraoperatively to assist with the implementation of the device programming. The relaxation programming will run for the duration of the operative procedure. At any time, the patient or anesthesia provider can decline to continue with the VR programming. At the end of the procedure the VR headset will be removed and standard postoperative care will commence.

Control Group

Subjects in the control group will be asked to refrain from using a VR modality intraoperatively. They will otherwise prepare for surgery and undergo perioperative care according to the current standard of care.

Follow Up

Postoperative care, including management in the PACU, will be conducted to the current standards of care. Data on postoperative outcomes, medications, and pain scores will be collected as indicated below in order to assess secondary outcomes.

After surgery, members of the study team will meet with the patient and ask them to complete a survey about their experience and overall satisfaction with the study. The survey will be built and administered using REDCap. If the patient is unable to complete the survey in-person, a link directly to the REDCap survey will be emailed to them.

Consistent with the current standard of care, patients will return to their surgeon's office for a postoperative visit, which typically occurs one month after surgery. Data from this visit, including surgical outcomes and assessment, will be passively abstracted from their medical record.

Data Collection

In order to assess our primary and secondary outcomes, data will be collected on intraoperative propofol dose, length of

PACU stay, as well as postoperative analgesia administration and pain scores. In addition, study team members will review the patients' medical records to collect demographic data and surgical characteristics including intraoperative vital signs, medication administration, and postoperative outcome data. Postoperative pain scores (i.e. 'Rate your pain on a scale from 0 to 10') are documented routinely as part of standard postoperative clinical care, and will be recorded at admission to the PACU and at PACU discharge. Additional baseline characteristics including but not limited to age, race, gender, BMI and comorbidities will be abstracted from the medical record. REDCap will be utilized for survey administration, data collection and storage.

Safety Monitoring and Reporting
This patient population is undergoing surgery and it is expected that they may have a number of unrelated adverse health events during their hospital course. Therefore, we will limit the scope of AE monitoring and reporting to the following:

- All Serious Adverse Events believed to be related to the study procedures
- All non-serious Adverse Events believed to be related to the study procedures

Adverse events will be monitored until the time of PACU discharge.

B. Statistical Consideration**s**

Sample Size Justification: Using a two sided α of 0.05, 80% power, and mean propofol doses of 0.069 (standard deviation 0.03) and 0.042 (SD 0.021), we anticipate needing to enroll a total of 32 patients to be able to detect a 50% reduction in the mean total intraoperative propofol dose. We believe this to be a reasonable effect size given preliminary data from a related study in which investigators were able to demonstrate a >50% reduction in dose for major joint surgery (Chan 2017). In order to compensate for anticipated drop out, we will plan to enroll an additional 8 patients to bring the total planned enrollment to 40 patients, with 20 in each of the two groups.

Data Analysis: Patients will be prospectively randomized using 1:1 block randomization of equal sizes. Analyses will be conducted using SAS version 9.3 (SAS Institute, Cary, North Carolina) or later. Descriptive statistics of the data will be performed. Continuous data will be represented using mean (± standard deviation) or median (interquartile range) for variables not normally distributed and compared using parametric or non-parametric t-tests as appropriate. Categorical data will be presented using proportions and compared using a chi-square or Fishers Exact test.

Primary Outcome
The primary outcome of this study is total intraoperative propofol dose, evaluated as a continuous variable. This will be assessed using a t test or Wilcoxon Rank Sum test, depending on the normality of the data. Although randomization should account for potential difference between groups, we will employ univariate and multivariable linear regression modeling to adjust for differences that persist, as necessary. Odds ratios and 95% confidence intervals will be reported. Our primary analysis will be conducted using intention to treat principles.

Secondary Outcomes
A priori secondary outcomes include length of PACU stay, postoperative analgesia dose requirements, postoperative pain scores, and overall patient satisfaction. Differences in time, medication administration and pain scores will be expressed as continuous variables. Patient satisfaction will be assessed from the REDCap survey, and will provide quantifiable estimates of satisfaction in multiple areas including but not limited to pain, anxiety, nausea, and overall experience. Adherence to the randomization assignment will be evaluated, and in the event that any patient receives an intervention different than their randomization assignment, we will also perform a per protocol analysis as an additional secondary outcome. For all secondary analyses, parametric or non-parametric tests will be employed in an analogous fashion to our baseline characteristics between groups.

C. Subject Selection

Inclusion Criteria:

1. Patients undergoing hand or wrist surgery at BIDMC under MAC

Exclusion Criteria:

1. Age < 18
2. Open wounds or active infection of the face or eye area
3. History of motion sickness
4. History of claustrophobia
5. History of seizures or other symptom linked to an epileptic condition
6. Patients who plan to wear hearing aids during the procedure
7. Patients with a pacemaker or other implanted medical device
8. Droplet or airborne precautions (as determined by BIDMC infection control policy)
9. Non-English speaking

Subjects will be enrolled without regard to race, gender or vulnerable category status. Non-English speaking patients will be excluded from this study as clear verbal communication during study procedures is essential. There is no reason to exclude pregnant women from this protocol. However, based on the study plan to recruit patients who will be undergoing an operation with anesthesia, we will be recruiting from a patient pool generally exclusive of women who are pregnant.

B4. POSSIBLE BENEFITS

As this study is the first to investigate the utility of VR as an adjunct to MAC anesthesia for hand and wrist surgery, we cannot guarantee any benefit to patients. It is possible that society will benefit from increased knowledge as to the utility of immersive relaxation VR techniques during surgery.

B5. POSSIBLE RISKS AND ANALYSIS OF RISK/BENEFIT RATIO

This study utilizes a non-invasive device therefore we expect the associated risks to be minimal. Potential risks of using virtual reality in this setting could include motion sickness, patient dissatisfaction, seizures and infection control concerns from shared equipment. To attempt to minimize the risk of motion sickness that can occur during virtual reality experiences, we have excluded patients with this known condition. Further, the anesthesia provider will be able to provide anti-emetics as prophylaxis or treatment based on their determination of clinical need. If a patient is dissatisfied with the VR experience during surgery, they will have the full ability to discontinue the intervention and receive standard anesthesia care at any time. Similarly, should the anesthesia provider determine that the clinical situation necessitates standard anesthesia practice, they will be able to discontinue use of the VR program at any time. Some people (about 1 in 4000) may have severe dizziness, seizures, eye or muscle twitching or blackouts triggered by light flashes or patterns, and this may occur while they are watching TV or experiencing virtual reality. This is most pronounced in children and the elderly. To mitigate this potential risk we are excluding children and patients with a history of seizures. If a patient experiences any symptoms the VR will be stopped and the patient will be treated accordingly. Lastly, to minimize the potential infection control risks from shared VR equipment, the primary investigator and the representatives from VR health have worked with the BIDMC infectious disease and infection control representatives to select equipment that can be wiped with sterilization wipes between uses. With the use of additional disposable barriers such as caps/hairnets, they are confident that the risk of transmission between subjects with the shared equipment to be minimal.

It is possible that by virtue of being enrolled in this study participants may receive fewer medications than they would otherwise receive if they did not participate. Risks associated with receiving less propofol include additional movement of the arm, temporary pain or discomfort during surgery, as well as the potential to experience discomfort or anxiety related to being alert in the operating room. If a patient experiences any pain or discomfort, the procedure will be temporarily paused and a local anesthetic injection can be administered. Of note, all patients will receive a nerve block as part of the standard of care for this surgery, regardless of study participation. This nerve block, when properly performed, renders the operative arm insensate and unable to move. Therefore, patients would only experience pain if this block was inadequate, further limiting the risk of experiencing pain. Additionally, participants will be monitored throughout the procedure by the clinical team. Need for additional medications will be consistently assessed throughout surgery, with assessment occurring independently of study participation and in the manner in which the response to surgical stimulation is usually assessed in standard anesthesia practice. .

As with any research study, there is the potential risk of a breach of confidentiality. To minimize the risk of dissemination of health information, all PHI from screening and enrollment will be saved on BIDMC research servers and only coded

information will be included in the REDCap database so as to prevent any unintended dissemination of PHI. No identifiable health information will be exchanged between the VRHealth company and the investigators.

We believe these risks to be outweighed by the benefit of knowledge gained about strategies that could potentially avoid unnecessary oversedation that can sometimes occur with MAC anesthesia.

B6. RECRUITMENT AND CONSENT PROCEDURES

Recruitment

Eligible patients will be identified by operative scheduling lists, surgical clinic visit schedules, and pre-admission testing (PAT) clinic visit scheduling lists. All patients who meet inclusion criteria with no exclusions will be approached in private areas for full written informed consent.

Consent

Once a patient is determined eligible, s/he will be approached by an IRB approved member of the study team. Informed consent will take place in the preoperative setting, either in a private area of a surgery clinic or PAT clinic. At the time of consent, all study procedures will be explained in detail, including the associated risks and benefits. The subject will have the opportunity to ask any and all questions, and will be reminded that participation is voluntarily and declining to participate will in no way affect their relationship with their health care provider or BIDMC. All subjects will be consented with curtains drawn or the door closed, assuring patient privacy. Written informed consent will then be obtained prior to initiation of any research procedures.

Please see Part A and the attached HIPAA waiver of authorization for additional information.

Subject Protection

It is unlikely that patients will be vulnerable to coercion or undue influence in this study. Although patients of one of the co-investigators (Dr. Rozental) will be approached for inclusion in this study, consent will be obtained from another member of the study team to ensure patients don't experience any undue influence. Patients will be informed that their decision to participate or not to participate will in no way affect their relationship with their health care provider, the performance or outcome of their upcoming surgery. Patients will also have the ability to discontinue their participation at any time.

B7. STUDY LOCATION

Privacy

All efforts will be taken to ensure patient privacy. Patient interactions including consent will take place in private clinical settings with curtains/doors closed so as to provide privacy and comfort. Throughout the study, only the minimum required information will be collected, assuring patient privacy during the study protocol as with usual patient care. Data collection from chart extraction will occur only on password-protected computers secured by the BIDMC firewall or in REDCap directly. Data collected will be limited to only the minimum necessary to accomplish the stated research purposes.

Physical Setting

Consent will be obtained from eligible patients in private clinical settings on the BIDMC East Campus, in either Dr. Rozental's hand surgery clinic or PAT clinic, with curtains/doors closed to ensure privacy. Study procedures and follow up will occur in the operating rooms on the East campus and at the surgery clinic, respectively. Data will be abstracted from the patients' chart and will be stored on password-protected computers behind the BIDMC firewall or in REDCap directly. All data collected on paper will be maintained in locked study offices accessible to only the study team. Study investigators have been trained to maintain PHI in a secure and compliant manner.

B8. DATA SECURITY

Data will be stored on password-protected computers behind the BIDMC firewall and entered into a computer database (REDCap). Computers and data collected on paper will be stored in locked study offices. For all analyses subjects will be identified only by their unique coded study ID number assigned for the sole purpose of this project. Limited information will be retained on patients who are prescreened and do not qualify, or who are approached and declined, for the purposes of generating a CONSORT diagram for the trial. At the completion of the study, final, aggregated data may be shared with VRHealth. No identifiable data will be shared.

B9 Multi-Site Studies

N/A – Single Center Study

Is the BIDMC the coordinating site? Yes ☐ No

Is the BIDMC PI the lead investigator of the multi-site study? Yes ☐ No

B10 Dissemination of Research Results

Please explain whether you will be able to thank subjects and provide research results and, if so, how this will be accomplished. If you do not think this is feasible, appropriate or applicable to this research, please specify why.

Patients will be thanked for their time throughout the study. There is no plan to share the data at the conclusion of the trial. Because study results are likely to be published a year or more after a given subject's participation, it is not feasible to send subjects follow-up with the published results. The study investigators are concerned that mailing the published manuscript and an additional thank-you note years after participation risks violating subject privacy, as mailing addresses are increasingly likely to change with passing time. It is out of the scope of this study to continue tracking mailing addresses after completion of enrollment since this is not a longitudinal study.
